# Round the Hospitals

**Published:** 1917-10-06

**Authors:** 


### ROUND THE HOSPITALS.

In a Note published in this section on page 495 of our issue for September 15 last, referring to the many War Office forms to be filled up in duplicate in the case of hospitals admitting wounded soldiers and others, we asked Sir Alfred Keogh please to note and if possible stop the duplication of forms. Further inquiries have convinced us that the forms in question are not those for which Sir Alfred Keogh is in any way responsible, and we offer him our apologies for the erroneous use of his name in this regard.

The winter session will soon be in full progress, and the active management of the College of Nursing is intimated by the notice of a meeting of the College of Nursing to be held in University College, Nottingham, on Monday afternoon, 8th instant, at 4.15, when Miss Rundle, the Secretary of the College, will give an address and explain in detail the objects and progress of the College of Nursing. This meeting is open to all nurses, and a cordial invitation is extended to Nottingham nurses and others whose work lies within a distance which will enable them to attend on the 8th instant in Nottingham. It is usual to expect that during the holidays both the work and the progress of a new movement like the College of Nursing must be suspended. No better evidence of the vitality and hold which the College of Nursing has properly won for itself with British nurses could be wished for than the fact that throughout the whole of the holiday period there has been a steady growth in the number of applications and eligible candidates for admission to the College Register.

One of the periodical meetings of the Scottish Matrons' Association was held in the Nurses' Memorial to King Edward VII. (Scotland), Edinburgh, on September 29, Miss Gill, R.R.C., President, in the chair. The number of members of this Association is now about 130, and Miss Gill has proved an ideal President by her regular and persistent attendance to the duties of the office and by the invaluable and continuous care she gives to everything calculated to promote the best interests of the members and the development and success of the Association. During war-time the meetings have frequently been interfered with owing to travelling difficulties, and the long distances represented by the fields of work covered by the members individually. We venture to suggest that it would be helpful to the members and tend to conserve their interest in the Association if a full agenda of the business to be transacted or the matters discussed at each meeting were to be sent by post to every member with the usual notice. At present we have found it difficult to follow the work of the Association owing to the absence of such a document and to the failure to record in this way the progress made with business arising out of the annual meeting.

One notable instance in this regard is the discussion which arose with reference to the Scottish Midwives' Act and the passing of a resolution petitioning the Board to establish immediately the Midwives' Roll to enable the Lord President of the Council to carry out the provisions of the Act by appointing two qualified midwives as members of the Board. We presume that this petition met with success, but we have had no evidence of the fact, nor any information as to whether the resolution of the Scottish Matrons' Association resulted in the postponement of the rules and regulations for the control and practice of midwifery in Scotland until after the appointment of the two midwife representatives.

The Scottish Matrons' Association is wholeheartedly interested in, and its members are supporters of, the College of Nursing. At the meeting on September 29 there was no special business, but the Chairman emphasised the source of encouragement and support which the members

**ROUND THE HOSPITALS—(*continued*).**

received from the regular meetings of the Association. Three annuitants of the Edith Cavell Memorial Fund are at present in the Home, and a fourth is about to enter it. Two small annuities are available and open to annuitants yet to be appointed. The name of the Scottish Nurses' Annuity Fund has been extended, and was altered on September 29 to that of "The Scottish Nurses' Benevolent and Annuity Fund." It was felt that the scope of usefulness of the Fund would be thus increased, and the members are encouraged by the intimation that a cheque for £25 and the promise of a yearly donation have been received on behalf of the Fund. An interesting discussion took place on the burning question of rations, which could not fail to fulfil a useful purpose. It was agreed to make arrangements for the holding of the annual meeting in Glasgow in February 1918. All Scottish matrons and nurses interested will obtain every information from the Honorary Secretary, Miss Graham, 15 Alva Street, Edinburgh.

AT a special meeting of the General Council of the Royal British Nurses' Association, held on September 27, to consider the amendments suggested by the Privy Council on the Draft Supplemental Charter, the following resolution was passed *nem. con.*: "The General Council of the Royal British Nurses' Association, after careful consideration of the objections raised by the Privy Council to the Draft Supplemental Charter, as agreed upon by the Corporation and the College of Nursing, Limited, have reluctantly come to the conclusion that it would not be to the interest of the Corporation to accept the alterations suggested."

MANY of our readers will be glad to hear how Miss E. Margaret Fox, the late matron of the Prince of Wales's General Hospital, Tottenham, has fared in Russia. She has been more than a year abroad, and shares Dr. Clemow's anxiety to remain in Russia should the necessity for the Unit's services still continue, which is uncertain at the moment. Since February last the Unit has been stationed at Ismail, where the members have had to attend to many hundreds of in-patients, in addition to others who come up for treatment. The aeroplane raids have often kept them extensively busy for considerable periods, and some cases have required much attention. At a review of troops in the Cathedral Square, which was attended with much pomp and circumstance, the Russian Government, for services rendered, decorated Dr. Clemow with the Order of St. Ann and Drs. Hind and Hime with the Order of St. Stanislau, whilst Miss Fox and the sisters received the Military Medal of St. George. Great interest was added to the occasion by the decoration of many of the soldiers present. Miss E. Margaret Fox's experience has included many trying positions, one of the latest being a bombardment by the enemy with some special long-distance guns, which resulted in shells falling in the town for two hours or more. Fortunately, none of the doctors or nurses was injured, nor were the hospital buildings or tents in any way damaged, though there were some narrow escapes. Miss Fox declares that Russian soldiers make good patients, and some of them bear unspeakable sufferings without complaint. The raid inflicted many terrible injuries, which were borne with exemplary patience. In thanking Miss Fox for her interesting notes, we tender her and her comrades the sympathy and good wishes of all her fellow-workers in the homeland.

DESPITE Miss Fox's experience of the Russian soldier as patient, the spirit of revolution appears to have penetrated to the hospital wards in some parts of Russia. According to the *Daily Telegraph's* Petrograd correspondent the Russian soldier since the revolution has changed from the most modest, contented, and thankful of patients to the most arrogant, exacting, and ungrateful. Wounded soldiers now, as a rule, drop all the old courtesies of address and issue commands to the nurses of the Red Cross "Sisters of Mercy" in place of what formerly would have been mild requests. Men employed by the Medical Service to do the menial work of the hospitals have compelled the nurses to scrub the wards and perform even more humble tasks; the patients have often insisted that the nurses should eat out of the same bowl with them. The large exodus from the Red Cross is attributed to these causes, 8,000 nurses having resigned to the end of July out of 40,000 who had been working for it at the time of the revolution.

WITH a view to secure an efficient midwifery service in every part of the country, with adequate fees for the midwives, the Association for Promoting the Training and Supply of Midwives have formulated proposals for a State-aided midwifery service in England and Wales. These proposals are generally approved by Queen Victoria's Jubilee Institute for Nurses and the Incorporated Midwives' Institute as a basis for necessary legislation. They include the payment of an adequate fee (25s. or 20s. according to the service rendered) to every midwife attending a confinement, the requisite sum for paying this fee to be provided from the Exchequer and not left to be met from such local rates as the local authority may be persuaded to levy, with some help from the Exchequer. The guaranteed fee to be available for any doctor, equally with any midwife, who undertakes to and does actually give the proper attendance and services at the confinement. It is considered desirable that attempts should not be made to recover from the patient any charge for the services rendered under the new scheme. The Exchequer money to be disbursed through an efficient local body which must supervise the systematic provision of midwives for the area, and the scheme to include and be conditional upon the provision of greatly improved arrangements for the inspection of midwives. It is estimated that a sum of about £1,000,000 per annum will be required in England and Wales for the carrying out of the scheme, which is intended to secure efficient midwifery services for every confinement where the income is below the £160 limit.

**For Nursing Affairs in Ireland, see p. 14; Training in Massage, p. 19; Matrons' and Sisters' Appointments, p. 20.**

